# High Incidence of Non-Random Template Strand Segregation and Asymmetric Fate Determination In Dividing Stem Cells and their Progeny 

**DOI:** 10.1371/journal.pbio.0050102

**Published:** 2007-04-17

**Authors:** Michael J Conboy, Ariela O Karasov, Thomas A Rando

**Affiliations:** 1 Department of Neurology and Neurological Sciences, Stanford University School of Medicine, Stanford, California, United States of America; 2 Geriatric Research, Education and Clinical Center, Veterans Administration Palo Alto Health Care System, Palo Alto, California, United States of America; 3 Neurology Service, Veterans Administration Palo Alto Health Care System, Palo Alto, California, United States of America; Baylor College of Medicine, United States of America

## Abstract

Decades ago, the “immortal strand hypothesis” was proposed as a means by which stem cells might limit acquiring mutations that could give rise to cancer, while continuing to proliferate for the life of an organism. Originally based on observations in embryonic cells, and later studied in terms of stem cell self-renewal, this hypothesis has remained largely unaccepted because of few additional reports, the rarity of the cells displaying template strand segregation, and alternative interpretations of experiments involving single labels or different types of labels to follow template strands. Using sequential pulses of halogenated thymidine analogs (bromodeoxyuridine [BrdU], chlorodeoxyuridine [CldU], and iododeoxyuridine [IdU]), and analyzing stem cell progeny during induced regeneration in vivo, we observed extraordinarily high frequencies of segregation of older and younger template strands during a period of proliferative expansion of muscle stem cells. Furthermore, template strand co-segregation was strongly associated with asymmetric cell divisions yielding daughters with divergent fates. Daughter cells inheriting the older templates retained the more immature phenotype, whereas daughters inheriting the newer templates acquired a more differentiated phenotype. These data provide compelling evidence of template strand co-segregation based on template age and associated with cell fate determination, suggest that template strand age is monitored during stem cell lineage progression, and raise important caveats for the interpretation of label-retaining cells.

## Author Summary

For each chromosome, the complementary DNA strands consist of a “younger” strand synthesized during the most recent round of DNA replication and an “older” strand synthesized during a previous cell division. When the strands separate to serve as templates for DNA synthesis during a subsequent round of replication, the two sister chromatids formed thus differ in terms of the template strand age. The “immortal strand hypothesis” predicts that a stem cell is capable of distinguishing between chromatids based on template age: when it divides, the self-renewing daughter will inherit the chromatids with the older templates, whereas the daughter destined to differentiate will inherit those with the newer templates. However, in vivo evidence in support of this hypothesis has been sparse. By labeling newly synthesized DNA in sequential divisions of stem/progenitors during muscle regeneration, we observed that almost half of the dividing cells sorted their chromatids based on template age. The more stem-like daughter inherited chromatids with older templates, and the more differentiated daughter inherited chromatids with younger templates. We propose that this phenomenon is a characteristic of asymmetrically dividing stem cells and their progeny.

## Abstract 3

Analysis of the segregation of older and younger DNA template strands in proliferating muscle stem cells provides compelling evidence of co-segregation based on template age and associated with cell fate determination.

## Introduction

How stem cells maintain genetic and epigenetic constancy throughout repeated divisions is currently unknown. According to the “immortal strand hypothesis” [1], as the stem cell divides asymmetrically, it selectively retains those sister chromatids containing the older template DNA strands in the daughter destined to be the renewed stem cell, thus passing the younger strands (with any mutations acquired during replication), to the tissue-committed cell. This phenomenon of template strand segregation was originally based on observations in embryonic fibroblasts [2] and supported by evidence from dividing cells in the intestinal epithelium [3]. Little additional evidence in support of this hypothesis was reported until recently when the immortal DNA hypothesis was revisited, and evidence in support of this process was detected in vitro in immortalized mouse tumor cells [4] and neurosphere cultures [5], and in vivo in intestinal [6], mammary [7], and muscle [8] stem cells. However, the in vivo examples of strand segregation have been limited to at most a few percent of the cells. Thus the phenomenon has yet to be broadly accepted and is attributed to a curious, but minor, cell population.

In studies (unpublished data) of the timing of proliferation and renewal of skeletal muscle stem cells, or “satellite cells,” we used different halogenated thymidine analogs (bromodeoxyuridine [BrdU], chlorodeoxyuridine [CldU], and iododeoxyuridine [IdU]) delivered at different times during regeneration to label sequential cell divisions. To our surprise, although proliferating cells incorporated both labels when we sequentially delivered two of the analogs, approximately half of the cells that ultimately returned to quiescence contained only the second label. Theoretically, this could be explained by the ability of the self-renewing cells to selectively retain the sister chromatids with the older, unlabeled template strands, consistent with the immortal strand hypothesis [1]. We thus examined myogenic progenitors during regeneration for direct evidence of segregation of older and newer template strands.

## Results

### An Unexpected Number of Regenerating Muscle Progenitor Cells Segregate Template DNA

**Figure 1:**
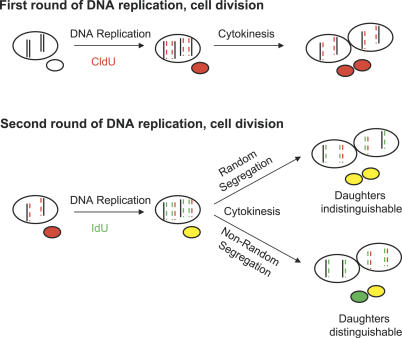
Models of Template Strand Segregation during Cell Division Models of random versus non-random segregation of template strand DNA. Only two pairs of chromosomes are shown for clarity. Newly synthesized DNA strands are indicated by color and dashed lines. At the first division, the newly synthesized DNA is labeled with CldU, is complementary to the template DNA, and thus is inherited equally. For the next round of DNA synthesis (during which the newly synthesized strand is labeled with IdU), one template strand is the older, unlabeled strand, and the other is the younger, CldU-labeled strand. If the cell segregates the chromatids with the older templates (and their complementary IdU-labeled strands) to one daughter, then all of the chromatids containing the younger, CldU-label templates would be inherited by the other daughter (also along with their complementary IdU-labeled strands). Note that this asymmetric inheritance using pulsed labels is only detectable after the second division when the strands labeled during a previous round of DNA replication have become templates for a subsequent round of DNA replication and inheritance.

**Figure 2:**
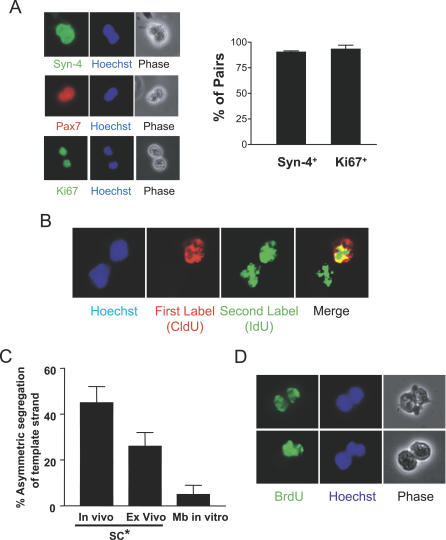
Evidence of Co-Segregation of DNA Template Strands during Muscle Progenitor Cell Division (A) Following muscle injury, CldU was administered to coincide with an early round of satellite cell division (~day 2 after injury), and IdU was administered to coincide with the subsequent round of DNA replication in the population. Cells were then isolated from muscle and plated singly on coated chamber slides, allowed to complete the current cell division, and then fixed. Cell pairs were analyzed for the expression of Syndecan-4 and Pax7, myogenic lineage markers [9,27], and for the marker of cell proliferation, Ki67. Representative pairs are shown on the left. The percentages of pairs that were myogenic (Syndecan-4^+^) and proliferating (Ki67^+^) were quantified and are presented graphically on the right. (B) Cell pairs were immunostained for CldU and IdU. Shown is a representative photograph of an immunostained pair of cells, in which both daughter cells were labeled with the second label, IdU (green), but only one daughter inherited the first label, CldU (red). (C) Quantification of asymmetric pairs. Almost half of the pairs derived from cells isolated from regenerating muscle showed asymmetric CldU staining as in (A) (all had symmetric IdU staining), and almost one third of the pairs of satellite cell progeny activated ex vivo in bulk myofiber explant cultures showed asymmetric inheritance of DNA label. Only a small percentage of pairs derived from established myoblast cultures (Mb in vitro) showed asymmetric inheritance of DNA label. Data represent mean ± the standard deviation (SD) (*n* = 4–7). SC*, progeny of activated satellite cells. (D) Muscle was injured and labeled with BrdU on day 2 of regeneration, as with CldU above (C). On day 3, the satellite cell progeny were prepared, plated sparsely, and cytochalasin D was added to arrest cytokinesis. The top row shows a cell pair symmetrically inheriting BrdU-labeled DNA, and the bottom row shows an asymmetric pair. Similar percentages of cells showed asymmetric inheritance of template strands using this protocol as in (C).

Muscle was injured to induce regeneration, and 2 d later, pulses of CldU were administered followed by pulses of IdU approximately 12 h later. As such, cells were labeled with CldU during one replicative cycle, and with IdU during the subsequent round of DNA replication (Figure 1). Cells were then isolated, plated singly, and after allowing a short time for individual cells to complete mitosis, the cells were fixed and immunostained for CldU and IdU. The cell pairs were clearly of the myogenic lineage because this procedure yields cell pairs that are nearly all positive for Syndecan-4 and Pax7, well-established myogenic markers [9], and the pairs are clearly replicating as demonstrated by expression of Ki67 (Figure 2A). Daughter cell pairs were analyzed for the distribution of the two labels. Nearly all the pairs of cells were labeled with IdU, confirming that they had undergone DNA replication during the more recent IdU pulse. However, strikingly, we observed asymmetric inheritance of CldU, with all of the detected label in only one daughter cell (Figure 2B), indicating that during the final cell division, one daughter cell had excluded those chromatids containing the template DNA that was labeled during the earlier cell division (see Figure 1). Even more striking was the fact that this was not a rare event whatsoever; this occurred in nearly half of the pairs (Figure 2C). We also examined the inheritance of labeled DNA in an ex vivo system in which satellite cells activate and proliferate while still associated with individual muscle fibers in culture [10,11]. We again observed a very high frequency of co-segregation of labeled chromatids (Figure 2C). By contrast, when the same labeling procedure was applied to proliferating myoblasts, asymmetric segregation of label was seen in only 5% of pairs (Figure 2C). Although markedly less frequent than in satellite cells activated in vivo or ex vivo, this still reflects a mechanism that is maintained in myogenic cells throughout replicative expansion, perhaps by the presence of the few stem-like cells that are propagated in myoblast cultures [12,13]. This suggests that the mechanisms underlying template strand segregation may be most active in cells maintained in the stem cell niche.

As a further confirmation that activated satellite cells asymmetrically segregate chromatids based on template age, we used a single-label protocol. Muscle was injured and BrdU was injected early during regeneration, comparable to the timing of the CldU pulse in the previous studies. The following day, after cells that had incorporated BrdU would have divided, giving rise to two BrdU-labeled daughters, cells were isolated, plated singly, and treated with either cytochalasin D or nocodazole for several hours to arrest cytokinesis. The cells were fixed and immunostained for BrdU to test for asymmetric segregation of the BrdU label. Of the pairs showing any BrdU label, again about half clearly showed only one daughter cell inheriting the BrdU (Figure 2D).

### Older Templates Segregate with the Less Differentiated Daughter

**Figure 3:**
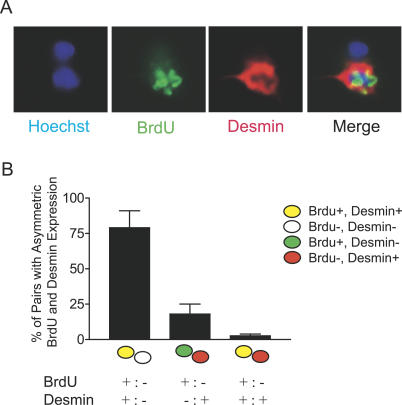
Divergent Cell Fates Associated with Asymmetric Segregation of Template Strands (A) Pairs of cells as in Figure 2 were co-immunostained for BrdU and the myoblast marker Desmin; representative images are shown. (B) The data from experiments as in (A) were quantified. Pairs with Desmin expression and asymmetric BrdU labeling were distinguished based on the pattern of Desmin expression (asymmetric and coincident with BrdU, asymmetric and mutually exclusive with BrdU, or symmetric). The legend for individual cells is shown to the right, and the legends for the cell pairs are shown below. Data represent mean ± SEM (*n* = 4).

According to the immortal strand hypothesis, the daughter cell inheriting the older template DNA is the renewing stem cell, whereas the other daughter acquires a more differentiated phenotype. However, our experimental protocol was not designed to test specifically for self-renewal, but rather focused on the proliferative expansion and myogenic lineage progression of the stem cell progeny. During myogenic lineage progression, satellite cells differentiate into fusion-competent, Desmin-expressing myoblasts [14,15]. We examined cell pairs exhibiting asymmetries in inheritance of DNA templates for the expression of Desmin to test if one daughter cell of each pair was more differentiated than the other and if specific templates segregated with specific cell fates. Pairs that showed asymmetric BrdU staining and any evidence of Desmin expression were further characterized and quantified. Strikingly, the great majority of pairs (79%) showed Desmin expression only in the daughter inheriting BrdU-labeled templates (Figure 3A and 3B), indicating a direct correlation between the inheritance of the younger template and the acquisition of a more differentiated fate, consistent with the underlying assumptions of the immortal strand hypothesis. Much smaller percentages of such pairs showed either asymmetric Desmin expression, but with the Desmin expressed in the BrdU-negative cell (18%), or symmetric Desmin expression (Figure 3B). This suggests that template strand co-segregation may not be limited to stem cell self-renewal, but may in fact occur more generally during stem cell expansion when asymmetric cell divisions or divergent daughter cell fates are determined.

Of the pairs in which BrdU-labeled templates were symmetrically inherited (about 50% of total cell pairs), nearly all were also symmetric for Desmin expression, either both positive (59%), reflecting symmetric divisions of myoblasts, or both negative (31%), reflecting symmetric divisions of early progenitors (Figure S1). Only a very small minority of pairs (9%) with symmetric BrdU showed asymmetric Desmin expression (Figure S1).

**Figure 4:**
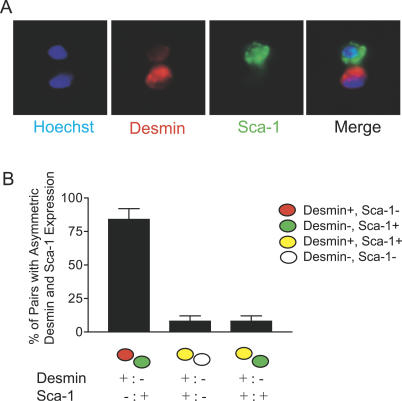
Segregation of the Oldest Template Strands to the Less Differentiated Progeny (A) Cells pairs were co-immunostained for Sca-1 and Desmin. Shown is a representative pair of cells demonstrating Sca-1 expression in one daughter and Desmin expression in the other. (B) The data from experiments as in (A) were quantified. Pairs with Sca-1 expression and asymmetric Desmin staining were distinguished based on the pattern of Sca-1 expression (asymmetric and mutually exclusive with Desmin, asymmetric and coincident with Desmin, or symmetric). The legends for the cells are as described for Figure 3B. Data represent mean ± S.E.M. (*n* = 4).

Several studies have identified Sca-1 as a marker of undifferentiated progenitors derived from skeletal muscle, demonstrating that satellite cells can give rise to progeny that express Sca-1 at least transiently [16–18]. Because of the treatments needed to detect both BrdU and Desmin immunohistochemically, we were not able to detect Sca-1 in populations that were also stained for both Desmin and BrdU. We could, however, detect clear Sca-1 immunostaining under milder conditions and compare its expression with Desmin in cell pairs. Given the strong correlation between asymmetric Desmin and asymmetric BrdU staining (Figure 3A and 3B), we used asymmetric Desmin as a surrogate marker of asymmetric inheritance of labeled template strands. Among pairs with asymmetric Desmin, the vast majority (84%) also showed asymmetric Sca-1 expression, with Desmin and Sca-1 being mutually exclusive (Figure 4A and 4B). This finding is consistent with the immortal strand hypothesis prediction that the cell inheriting the older template (in this case, the Desmin^−^ cell) is the more undifferentiated cell as reflected by the expression of Sca-1. Only very rarely were pairs detected in which Desmin was expressed asymmetrically and Sca-1 was expressed (whether asymmetrically or symmetrically) in the Desmin^+^ cell (Figure 4B). Virtually all pairs expressing Desmin symmetrically did not express Sca-1 (Figure S2), consistent with high Desmin expression specifying a more differentiated myoblast and Sca-1 expression reflecting a more immature progenitor.

**Figure 5:**
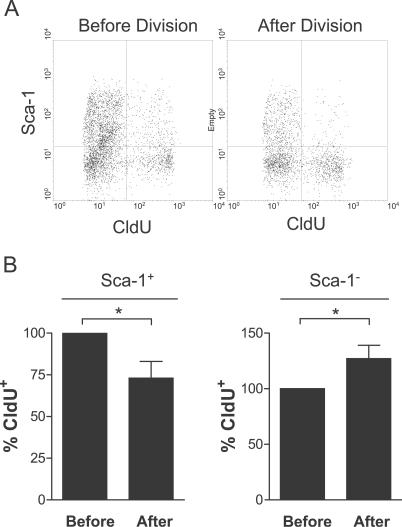
Correlation of Template Strand Segregation with Cell Fates in Myogenic Progenitors Cells were labeled and isolated from regenerating muscle as in Figure 2. Half of the cells were fixed immediately (Before Division). The other half (After Division) was cultured overnight to allow the cells to complete the current cell cycle and divide. The cells were then harvested and analyzed by FACS for Sca-1, the labeled (younger) template (CldU), and newly synthesized DNA (IdU). Gatings were on forward scatter (FSC) and IdU-labeled cells. (A) A representative FACS plot of Sca-1 versus CldU is shown in the population before and after cell division. (B) Quantitation of FACS plots as shown in (A) demonstrates the decrease in the percentage of Sca-1^+^ cells that have labeled template strands before and after cell division and the corresponding increase in the percentage of Sca-1^−^ cells having labeled template strands (*n* ≥ 3; a single asterisk [*] indicates *p* < 0.01).

Using these paired cell assays, our data are thus supportive of the immortal strand hypothesis and suggest that template strand segregation is occurring in a large percentage of satellite cell progeny coincident with cell fate decisions. The more immature, Sca-1^+^ cells undergo asymmetric divisions in which the oldest (unlabeled, in our studies) templates segregate to the daughter that retains the less differentiated phenotype as reflected by Sca-1 expression. The other daughter, by contract, acquires the newer templates (labeled, in our studies, with CldU in the double-label experiments [Figure 2] or BrdU in the single-label experiments [Figure3]) and adopts a more differentiated phenotype as reflected by Desmin expression. These results would predict that the percentage of Sca-1^+^ cells that are also CldU^+^ (using the double-label protocol) would decrease through a round of cell division, whereas the percentage of Sca-1^−^ cells that are also CldU^+^ would increase. To test this directly, we performed experiments as in Figure 2, but at the time of isolation, half of the cells were fixed immediately as a “before division” snapshot of the population. The other half was cultured for an additional 12 h before harvest and fixation as the “after division” population. Cells were then immunostained for Sca-1, CldU (the presumed younger template), and IdU (incorporated into the complimentary DNA strands of divided cells), and analyzed by fluorescence-activated cell sorting (FACS). The proportion of cells expressing Sca-1 and labeled with CldU decreased substantially during this time, whereas the Sca-1^−^, CldU^+^ proportion increased by a corresponding percentage (Figure 5). This is consistent with parental Sca-1^+^ cells segregating older (unlabeled) and younger (CldU-labeled) templates into two daughters that acquire different fates. These results are also consistent with the proportions of cells asymmetrically inheriting template strands and expressing Sca-1 that we observed in the paired cell assays above.

## Discussion

We propose a model of muscle stem cell proliferation in which muscle progenitor cells divide asymmetrically to generate both myoblasts and immature, undifferentiated cells (some of which are likely destined to return to quiescence as replacement satellite cells in vivo), and symmetrically in order to expand either the pool of progenitors or fusion-competent myoblasts necessary to promote effective muscle repair. The finding of asymmetric inheritance of template strands in the case of the asymmetric divisions and the association of the older templates and the more undifferentiated phenotype is compelling evidence in support of the immortal strand hypothesis, but extends the association of template strand co-segregation to a much broader range of stem cell lineage decisions than just self-renewal. Mechanistically, our data suggest that there must be an ongoing monitoring of template strand age and a process to segregate those strands according to age in a sequential manner, not merely the existence of one immortal strand.

The extraordinarily high frequency of muscle progenitor cells exhibiting template strand segregation during muscle regeneration, as opposed to the low frequencies observed in other in vivo systems [6,7], promises to make this system valuable to study mechanisms of asymmetric inheritance of DNA template strands. The high frequency observed in our in vivo studies may relate to the fact that we analyzed cells for asymmetric inheritance of template strands during the process of tissue repair, whereas other in vivo studies have sought evidence of this process during normal homeostatic turnover of tissues [6,7].

In addition to providing strong support for the immortal strand hypothesis and expanding the scope of that hypothesis, the findings presented here have additional important implications. First, the assessment of the proliferation kinetics of stem and progenitor cells has been carried out in many tissues by analyzing the dilution of label incorporated into DNA [19,20]. The ability of stem or progenitor cells to segregate all label to only one daughter would clearly confound the interpretation of all studies that have heretofore assumed equivalent distribution of label to daughter cells and a simple geometric relationship between label dilution and replicative history. Second, our data require a careful analysis of the use of “label retention” to identify stem cells in tissues, based on the assumption that label retention is equated with very long cell cycle times or quiescence [21–24]. Rather, our data suggest an alternative process by which a cell, even a rapidly dividing cell, could take up label and generate label-retaining progeny. If labeled chromatids continue to co-segregate through repeated rounds of DNA replication and cell division (see Figure 1), then label-retaining cells can, theoretically, be maintained indefinitely. Accordingly, such a cell would have an indeterminate replicative history since the time the label was administered. The other implication of this caveat is that a label-retaining cell could represent any stage along the lineage from the most undifferentiated stem cell to the most differentiated progeny. In our studies, the label-retaining cell was, in fact, the more committed of the two daughters, and the label-excluding cell was the more undifferentiated of the two. Clearly, the mechanisms that result in label-retaining cells in any tissue may be more complex than simply long cell cycle times, and the relationship between label retention and stage of differentiation may likewise vary from tissue to tissue and under different biological contexts.

## Materials and Methods

### Antibodies and immunostaining.

Mouse antibody clone B44 recognizing IdU (and also BrdU) was obtained from BD Biosciences (San Diego, California, United States); rat antibody clone BU1/75 (ICR1) recognizing CldU (and also BrdU) and rat anti-Sca-1 were from Novus Biologicals (Littleton, Colorado, United States). Mouse and rabbit anti-Desmin antibodies were purchased from Sigma (St. Louis, Missouri, United States) and used at 1:200. Mouse hybridoma supernatant anti-Pax7 was from the Developmental Studies Hybridoma Bank (http://www.uiowa.edu/~dshbwww/) and used at a dilution of 1:5. Chicken IgY anti-Syndecan-4 was a generous gift from Dr. Brad Olwin (University of Colorado) and was used at 1:3,000. Rat anti Ki67 was from DakoCytomation (Glostrup, Denmark) and was used at 1:50. Isotype-matched antibodies were used as controls. Antibody staining was performed as previously described [15]. Unless otherwise indicated, primary antibodies were used at 0.5–1 μg/ml. Higher concentrations resulted in detectable cross-reactivity for the antibodies against the halogenated thymidine analogs. Secondary antibodies were Alexa 488- or 546-coupled anti-rat, anti-rabbit, anti-chicken, or anti-mouse antibodies (Invitrogen/Molecular Probes, Carlsbad, California, United States) used at 1:2,000 for immunofluorescence microscopy.

For detection of labeled DNA, cells were fixed in 70% ethanol, washed in PBS, denatured in 2.5 M HCl for 30 min, and permeabilized in 0.25% Triton-X-100 for 5 min before incubation with primary antibodies overnight in PBS/5% fetal bovine serum. For Pax7 detection, cells were fixed in paraformaldehyde and incubated as above with Triton. For Sca-1 and Syndecan-4 labeling, cells were fixed in 4% paraformaldehyde, washed, and incubated with primary antibody (in PBS/5% fetal bovine serum for Sca-1; and in 10% BlockHen [Aves Labs, Tigard, Oregon, United States] for Syndecan-4) overnight without any detergent permeabilization [17].

### Muscle injury model.

Muscle injury was induced by the injection of 1–2 μl of cardiotoxin I (100 μg/ml; Sigma) into 24 sites in muscles of the limb. This produces a diffuse necrotic injury and results in the activation of satellite cells throughout the muscles.

### Thymidine analogs and labeling.

BrdU, IdU, and CldU were purchased from Sigma and used at a dose of 30 mg/kg (subcutaneously). For CldU/IdU double-labeling experiments, two doses of CldU were administered 4 h apart, with the first dose administered 48 h after the muscle injury. Approximately 8 h after the second dose of CldU, IdU was administered also by two sequential injections, the second one 4 h after the first. For in vivo BrdU-labeling experiments, two doses were administered 4 h apart, with the first dose administered 48 h after the muscle injury. For in vitro experiments, thymidine analogs were used at a final concentration of 5 μM in the media. For CldU/IdU experiments, either in vivo or in vitro, similar results were observed when the two labels were reversed.

### Preparation of myogenic progenitors from muscle.

As previously described [25], muscle was dissected, digested in 0.25 U/ml collagenase type II (Sigma) in HEPES buffered media, and dissociated by trituration into fiber fragments. Fiber-associated cells were liberated either by further digestion in 0.5 U/ml dispase and 80 U/ml collagenase in media and then filtration and subsequent washing of cells in PBS, or by trituration in media through a 20-gauge needle [26]. Both methods gave similar results. Cells obtained by this methods are more than 95% positive for the myogenic cell markers CD34 and M-cadherin and less than 2% positive for the endothelial cell marker PECAM [18,25].

### Paired cell assay to assess for asymmetric inheritance of template strands.

Cells labeled in vivo and prepared as above were plated at a very low density (~10 cells/mm^2^) onto 4- or 8-well chamber slides coated with ECM gel (Sigma) diluted to 1:100. Satellite cells activated ex vivo in bulk myofiber explant cultures were labeled with a pulse for 8 h on day 2 after explantation, maintained in growth medium for an additional 12 h, and then liberated from the fibers and plated singly, as above, early on day 3. Established myoblast cultures (passage 20–30 after isolation as bulk cultures [25]) were labeled, plated singly, and analyzed identically. Direct microscopic examination revealed that sparsely plated cells adhered within about 1 h and that negligible cell migration occurred during the subsequent period before analysis of cell pairs. After cells were attached, cytochalasin D (2 μM final concentration; Sigma) or nocodazole (1 μM final concentration; Sigma) was added to block cytokinesis. Cells were fixed 2–4 h after plating, immunostained, and scored as a “divided pair” if they were within one cell diameter of each other, and more than 50 cell diameters away from other cells in the 20× field of view. Between 100 and 200 cell pairs were scored per experiment, and the number of replicate experiments is described in the figure legends.

### FACS analysis to assess for asymmetric inheritance of template strands.

For co-staining of Sca-1, CldU, and IdU for FACS analysis, Sca-1 was detected as above using Alexa 647 as the secondary antibody. The samples were then re-fixed, permeablized with 0.25% Triton-X-100, digested with DNAse1 in F-10 medium [8], and immunostained for CldU and IdU as above, using Alexa 488 or R-phycoerythrin anti-rat secondary antibodies, and PE- or FITC-conjugated mouse anti-BrdU (IdU) clone B44. Isotype-matched antibodies were used as negative controls and for gating. FACS acquisition was performed on a FacsCaliber model (BD Biosciences), and analysis was performed using WinMDI 2.8 software (Joseph Trotter, http://facs.scripps.edu).

## Supporting Information

Figure S1 Quantitation of Desmin Expression among Daughter Cell Pairs with Symmetric Inheritance of BrdU-Labeled Template Strands The data from experiments as in Figure 3A were quantified. Pairs with Desmin expression and symmetric BrdU labeling were distinguished based on the pattern of Desmin expression (both positive, both negative, or asymmetric). The legend for individual cells is shown to the right, and the legends for the cell pairs are shown below. Data represent mean ± standard error of the mean (SEM) (*n* = 4).
(18 KB PDF)

Figure S2 Quantitation of Sca-1 Expression among Daughter Cell Pairs with Symmetric Expression of Desmin The data from experiments as in Figure 4A were quantified. Pairs with symmetric Desmin expression were distinguished based on the pattern of Sca-1 expression (both negative, both positive, or asymmetric). The legend for individual cells is shown to the right, and the legends for the cell pairs are shown below. Data represent mean ± SEM (*n* = 4).
(16 KB PDF)

## Abbreviations

BrdU: bromodeoxyuridine
CldU: chlorodeoxyuridine
FACS: fluorescence-activated cell sorting
IdU: iododeoxyuridine
SEM: standard error of the mean
