# Correction: High Incidence of Non-Random Template Strand Segregation and Asymmetric Fate Determination In Dividing Stem Cells and their Progeny

**DOI:** 10.1371/journal.pbio.0050182

**Published:** 2007-07-17

**Authors:** Michael Conboy, Ariela Karasov, Thomas Rando

In *PLoS Biology*, volume 5, issue 5: doi: 10.1371/journal.pbio.0050102

The first author, Michael J. Conboy, should be listed as a corresponding author. His E-mail address is conboymj@berkeley.edu.

