# Supplementary material for: High Incidence of Non-Random Template Strand Segregation and Asymmetric Fate Determination In Dividing Stem Cells and their Progeny
Source: PLoS Biol. 2007 Apr 17;5(5):e102. doi: 10.1371/journal.pbio.0050102 (PMC1852584; doi:10.1371/journal.pbio.0050102)
Supplement: Supplementary file 1 [file pbio.0050102.sg001.pdf]

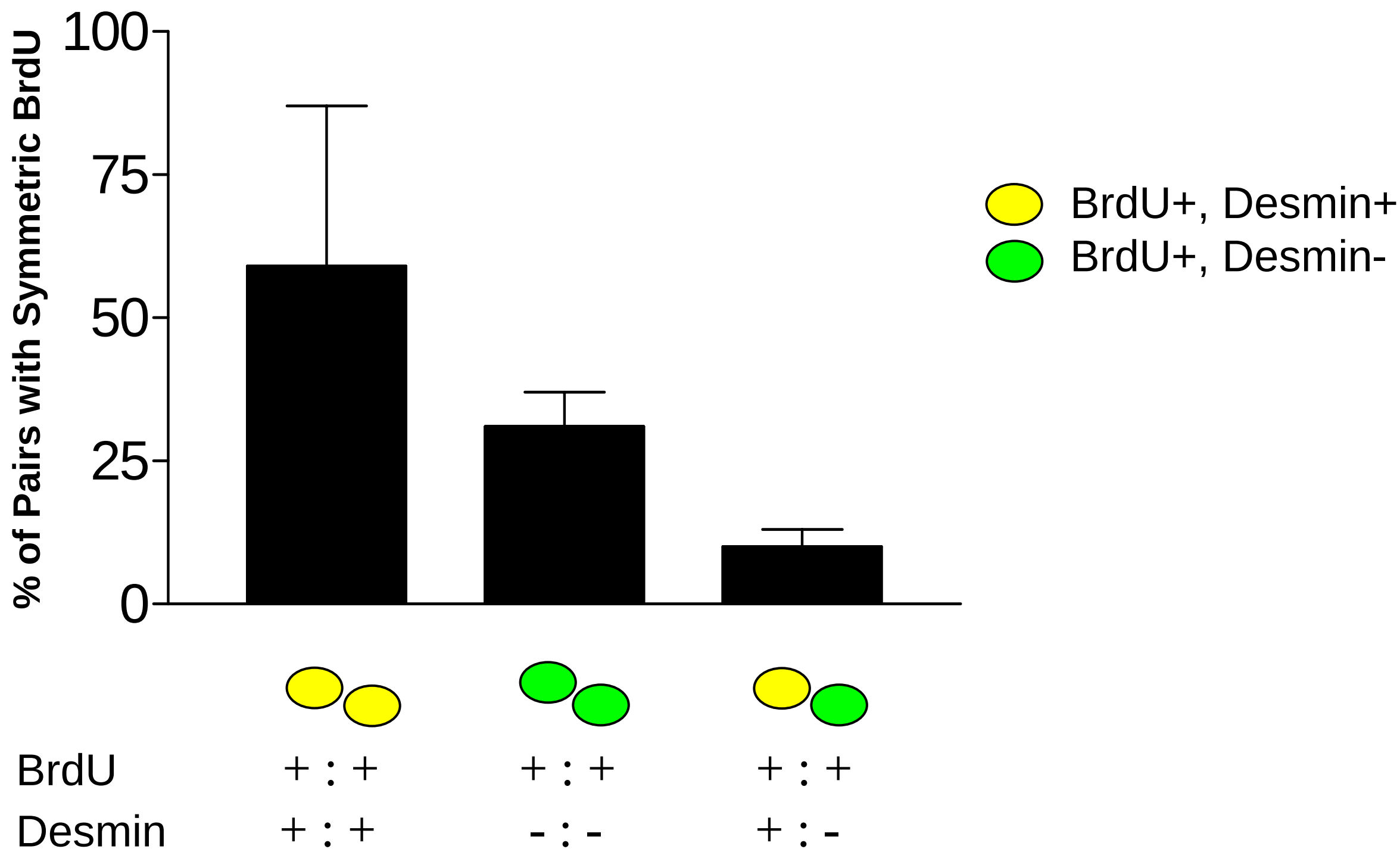

Figure S1. Quantitation of Desmin expression among daughter cell pairs with symmetric inheritance of BrdU-labeled template strands.

The data from experiments as in Figure 3A were quantified. Pairs with Desmin expression and symmetric BrdU labeling were distinguished based on the pattern of Desmin expression (both positive, both negative, or asymmetric). The legend for individual cells is shown to the right and the legends for the cell pairs are shown below. Data represent mean ± S.E.M. (n = 4).
