# Supplementary material for: High Incidence of Non-Random Template Strand Segregation and Asymmetric Fate Determination In Dividing Stem Cells and their Progeny
Source: PLoS Biol. 2007 Apr 17;5(5):e102. doi: 10.1371/journal.pbio.0050102 (PMC1852584; doi:10.1371/journal.pbio.0050102)
Supplement: Supplementary file 2 [file pbio.0050102.sg002.pdf]

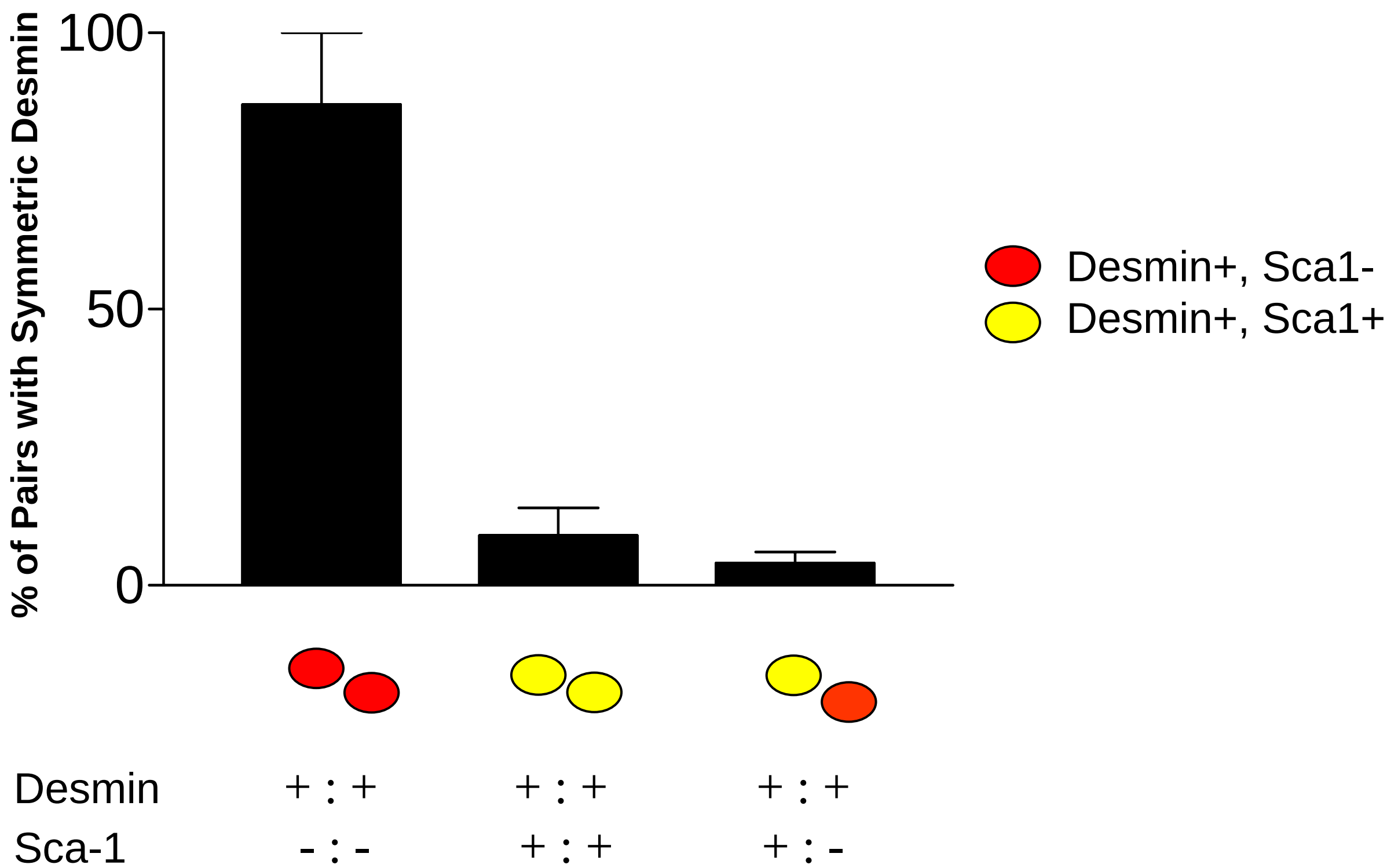

Figure S2. Quantitation of Sca-1 expression among daughter cell pairs with symmetric expression of Desmin.

The data from experiments as in Figure 4A were quantified. Pairs with symmetric Desmin expression were distinguished based on the pattern of Sca-1 expression (both negative, both positive, or asymmetric). The legend for individual cells is shown to the right and the legends for the cell pairs are shown below. Data represent mean ± S.E.M. (n = 4).
